# Cancer survivors’ experiences of a physical activity program in primary care: a qualitative study

**DOI:** 10.1007/s11764-024-01571-w

**Published:** 2024-03-22

**Authors:** Famke Huizinga, Eleonora A. M. Kieboom, Mathieu H. G. de Greef, Annemiek M. E. Walenkamp, Annette J. Berendsen, Marjolein Y. Berger, Daan Brandenbarg

**Affiliations:** 1 Department of Primary and Long-Term Care, University Medical Centre Groningen, University of Groningen, PO Box 196, FA 21, 9700 AD Groningen, The Netherlands; 2 Department of Human Movement Sciences, University Medical Centre Groningen, University of Groningen, PO Box 196, FA 23, 9700 AD Groningen, The Netherlands; 3 Department of Medical Oncology, University Medical Centre Groningen, University of Groningen, DA 11, PO Box 30.001, 9700 RB Groningen, The Netherlands

**Keywords:** Qualitative research, Exercise, Cancer survivors, Primary care

## Abstract

**Purpose:**

This study aimed to gain insight into the experiences of, and reasons for, cancer survivors participating in a primary care PA program.

**Methods:**

We interviewed 17 patients from 11 Dutch GP practices. Patients were selected by purposive sampling based on their general practice, gender, educational level, motivation for PA, and change in PA. Interviews were audio recorded, transcribed verbatim, and pseudonymized for inductive thematic analysis.

**Results:**

Three domains were identified with five themes: institutional domain: GP practice; program-specific domain: content sessions and PA, and activity tracker and goal setting; individual domain: experienced benefits, and personalized care needs. Participants valued the PA program because it was offered close to home, without additional costs, and by a trusted practice nurse familiar with the patients’ medical background. Activity tracker use and goal setting motivated many participants but also led to demotivation and feelings of failure in others. Reported benefits included behavior change and favorable health outcomes. Many patients expressed the need to personalize psychological support and the program’s timing.

**Conclusions:**

Access to a PA program in a primary care setting is valued for its accessibility and experienced health benefits, but also seems to meet an unmet need for support in picking up life during cancer recovery.

**Implications for Cancer Survivors:**

Primary care is important for continued care of cancer survivors. An accessible PA program in this setting may fulfil a need for not only lifestyle support but also continuing life after cancer treatment.

## Introduction

There is increasing evidence that physical activity (PA) is beneficial for patients living with and beyond cancer. It has the potential not only to improve physical functioning [1, 2] but also to alleviate fatigue [1, 3–5], depression [6–8], and anxiety [3, 9, 10]. Evidence-based guidelines therefore recommend PA programs both during and after cancer treatment [11, 12]. Despite the reported benefits, however, survivors generally have low uptake [13, 14] and do not achieve the recommended PA levels [15, 16]. Indeed, a patient’s PA level typically declines after a cancer diagnosis and fails to return to pre-diagnosis levels by up to 30 months after diagnosis [17, 18]. This may be related to the perceived barriers and facilitators among patients.

Patient-related barriers to participation include a lack of time, of motivation, or of confidence regarding PA, as well as the presence of symptoms related to the disease and its treatment, such as fatigue, pain, feeling unwell, and limited mobility [16, 19–21]. A recent qualitative study among cancer survivors found important barriers at an organizational level as well [22]. These included the program being offered at inappropriate place and time, a lack of knowledge, skills, and interest in PA among health care workers, non-involvement of the general practitioner (GP) in cancer care, and cost (e.g., insufficient insurance coverage) [22]. The authors conclude that a strategy directed at these organizational barriers would greatly facilitate the implementation of PA programs. Other studies likewise address the importance of improving the availability and accessibility of the PA program, as well as associated social support, counselling, or advice [19, 20].

In the Netherlands, oncology rehabilitation programs primarily operate within secondary care settings such as hospitals or rehabilitation centers. Primary care typically lacks a formal role in post-cancer care. This is despite its potential advantages such as proximity to patients, longstanding patient relationships, and experience in lifestyle consultations [23, 24]. Notably, cancer survivors perceive their GP as the appropriate health care professional to address lifestyle-related concerns [24] and demonstrate increased utilization of GP services during and after cancer treatment [23, 25, 26].

To tackle organizational barriers, we therefore implemented a PA program for cancer survivors in a primary care setting of Dutch GP practices [27]. Our program incorporates elements designed to improve participation of PA programs by cancer survivors [28], including individual counselling by a trained practice nurse, and using behavioral change techniques such as an activity tracker to set and evaluate goals, and allowing patients the autonomy to choose their preferred type and intensity of PA [27]. To evaluate and improve the PA program from a patient’s perspective, we performed a qualitative study to evaluate their reasons for, and experiences of, participation.

## Methods

### Design

We conducted a qualitative study based on a phenomenological framework, using semi-structured individual interviews with patients who took part in the PA program. The aims were (1) to explore the reasons for participation, (2) to identify positive and negative experiences with the PA program, and (3) to identify recommendations for improvement. We used the COREQ (Consolidated Criteria for Reporting Qualitative Research) checklist when reporting this study [29].

### Setting

Participants were recruited from the Stimulation of Daily Activities (SoDA) study, comprising 149 cancer survivors engaged in a PA program their own primary care setting: the GP practice [27]. In the Netherlands, every citizen is registered at a GP practice. GPs serve as gatekeepers to secondary care, with GP services covered by health insurance, unlike secondary care which requires patient deductibles. Approximately 80% of GP practices employ a practice nurse responsible for chronic care management, including diabetes, pulmonary disease, and cardiovascular disease [30], involving routine assessments and lifestyle consultations. Patients could participate in this study if they were at least 6 months after end of treatment, and did not participate in any other exercise or rehabilitation program. The PA program, called the COACH method, has shown to be effective in increasing PA in other patient groups before [31–36], and comprised six counselling sessions with the practice nurse over 9 months, during which patients were coached to increase their PA in their daily life at home. PA was not supervised. Patients wore an activity tracker for goal setting and feedback.

Practice nurses were all certified to work within GP practice and have completed extra training to administer the PA program. This training was conducted by experienced instructors (FH and MHG). The training encompassed theories and strategies of behavioral change [27], guidance on using the activity tracker, and role-playing exercises for the initial two sessions, with feedback provided by peers and/or instructors. Additionally, they received education from oncology nurses regarding common physical and psychosocial issues among cancer survivors. More details about the PA program and study design can be found elsewhere [27].

### Participants

We invited patients who participated in at least four sessions of the program (i.e., 3 months) and those who had discontinued the program after at least two sessions (i.e., 6 weeks). We used purposive sampling to maximize variation in the GP practices and in the patients’ age, sex, educational level, motivation for PA, and change in PA level (i.e., daily number of steps) from baseline to 3 months. We invited patients by telephone, and for those who showed an interest, sent them an information letter and an informed consent form to sign. Participants received a gift card of 20 euros.

### Data collection

Two researchers (FH and DB) developed a topic list in close collaboration with a research team consisting of a GP, practice nurse, human movement scientist, and two patient advocates from the Dutch Breast Cancer Society (Table 1). The main question of interest was: “What are your experiences with the PA program?”.

**Table 1:** Topic list*

What is the reason you decided to participate in the program?
Looking back, what are your experiences of participating in the program?
What are your experiences of the coaching sessions delivered by the practice nurse?
What are your experiences with the activity tracker?
How did you feel about setting up and evaluating the goals?^#^
What aspects of the program helped you in increasing your PA?^#^
What aspects of the program helped you to maintain the program?^#^
How did you experience the individualized set-up of the program without other peers being involved?^#^
What is your opinion about the GP practice offering the program?
Did you notice any changes in your health or well-being after participation compared to before?
Do you do things differently after participation in the program compared to before?
To what extent do you think the program is suitable for cancer survivors?
What would be the best timing in the disease trajectory to offer this program?
Did you think anything was missing from the program? If so, can you elaborate?
Can you think of any positive or negative things in the program? If so, what are these?
Do you have any advice about how to improve the program?

*All questions related to the PA program delivered to cancer survivors at their GP practice as part of the Stimulation of Daily Activities (SoDA) study.

^#^These topics were identified during early interviews and added to later interviews

*GP*, general practitioner; *PA*, physical activity

All interviews were conducted in the patient’s home or the University Medical Centre, depending on patient preference. DB performed the first interview and FH performed subsequent interviews. DB is an epidemiologist and trained interviewer with a doctoral degree in primary health care; FH is a psychologist and human movement scientist. The interviews were audio recorded, transcribed verbatim, and pseudonymized. Field notes were also created after each interview.

Patient age, gender, and educational level were collected by questionnaire, and disease characteristics were collected from GP medical records. Educational level was categorized into low (primary education or lower secondary education), medium (secondary or postsecondary education), and high (tertiary education) [37]. Patient motivation for PA was measured by the practice nurse at the start of the PA program using the stage of change score, which could range from 1 (no motivation to change) to 5 (high motivation for, or inclusion of, PA in daily routines) [38]. The practice nurse assessed the change in PA levels, which we defined as the change in the mean daily number of steps measured by the activity tracker from session one to four [27].

### Data analysis

Data collection and analysis occurred iteratively, allowing new topics to be added for upcoming interviews (Table 1). We used thematic analyses, as proposed by Braun and Clarke [39]. Two researchers (FH and EK) independently applied inductive coding to text segments relevant to the research question, and two patient advocates identified codes in a subset of six transcripts. The independently coded interviews were then discussed, after which final codes were assigned based on consensus. A third researcher (DB) was consulted in case of disagreement. Themes were identified by the researchers (FH, EK, DB) and patient advocates for discussion within the research team. We performed two more interviews when data saturation was suspected; if no new themes emerged, we assumed data saturation and conducted no further interviews. ATLAS.ti (Version 22) was used for data coding. Quotes were translated into English by a native speaker.

### Ethical considerations

The Medical Research Ethics Committee of the University Medical Centre Groningen, the Netherlands, concluded that the SoDA study was not subject to the Dutch Medical Research Involving Human Subjects Act (registration number: 201900586). Participants gave permission to contact them for any study-related questions and provided signed informed consent for this interview study.

## Results

### Participants

We invited 19 patients between August 2022 and May 2023, of whom 17 from 11 GP practices agreed to be interviewed (Table 2). The two patients not included in the interviews were not interested in participation: one felt he had already shared his experiences with the practice nurse, and one did not provide a reason. Participants ranged in age from 41 to 79 years and about half were female (59%). The interviews lasted 39 min on average (range, 26 to 56 min).

**Table 2:** Characteristics of the interview participants (*n* = 17)

Pt. No	Sex (M/F)	Age (Yrs)	Education	Cancer diagnosis	Years since diagnosis	Cancer treatment	PA motivation	No. sessions	PA Change
1	M	58	Medium	Gastro-intestinal	1	S	Moderate	4	− 1000
2	F	41	Medium	Breast	1	S, CT, RT, H	Low	3	+ 2979
3	F	45	High	Breast	2	S, CT, RT	Moderate	4	− 4000
4*	F	68	High	Breast	2	S, CT, RT, H	High	2	NA
5*	M	65	Medium	Colon	3	S	Moderate	2	NA
6	F	65	Low	Vulva	3	S, RT	High	6	+ 2186
7	M	77	Medium	Hematologic, lung	3	S, CT	High	4	+ 5000
8	F	55	Medium	Ovarium	4	S, CT	High	5	− 2593
9	M	59	Medium	Bladder	4	S	Moderate	5	+ 3014
10	M	73	High	Prostate	5	RT, H	High	6	NA
11	M	71	Medium	Prostate	5	RT, H	High	4	− 4000
12	F	67	Low	Kidney	8	S	Low	4	NA
13	F	69	Low	Breast	11	S, CT, RT, H	High	4	+ 4365
14*	F	79	Low	Melanoma	11	S	Moderate	5	− 3736
15*	F	61	Low	Kidney	13	S	Moderate	4	NA
16	M	69	Low	Hematologic	14	SCT, CT, RT	High	5	+ 8780
17	F	49	High	Hematologic	27	SCT, CT, RT	Moderate	6	+ 3514

Education: low = primary education or lower secondary education, medium = upper secondary education, high = tertiary education. Motivation for PA: 4, 5 = high, 3 = moderate, 1, 2 = low. * = dropouts. PA change is measured in steps

*CT* chemotherapy, *F* female, *H* hormone therapy, *M* male, *NA* not applicable, *No.* number, *PA* physical activity, *Pt*. patient, *RT* radiotherapy, *S* surgery, *SCT* stem cell transplant

### Reasons for participation in the PA program

Patients reported that they participated for reasons related to health, care, or research. Among the health-related motives, they reported taking part to improve PA, energy, or physical fitness levels. Among the care-related motives were patients’ desires to have contact with their GP after cancer treatment had ended or to receive professional assistance to increase or maintain PA levels. Also, several patients participated for research-related motives, stating that they wanted to help others by contributing to research.

### Themes

**Fig. 1:**
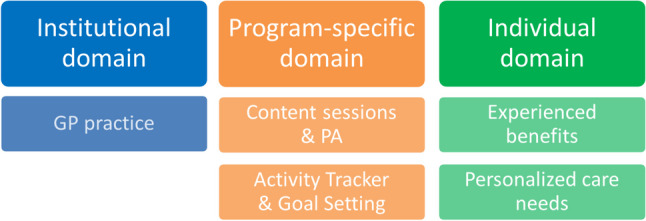
Code tree of the domains and themes

Three domains were identified with five themes: institutional domain: GP practice; program-specific domain: content sessions and PA, and activity tracker and goal setting; and individual domain: experienced benefits and personalized care needs (Fig. 1).

#### Theme 1: GP practice (institutional domain)

Many interviewed patients reported that they appreciated the GP practice providing the PA program because it was closer to their homes. They also mentioned the benefit that there were no costs related to participation in either the program itself or travel.

Patient 6, Female, 65Y:* I liked that it* [the PA program] *was here* [at the GP practice]*, because I did not have to travel to the city every time. That* [not being close] *might have held me back, and perhaps I would have said ‘I won’t participate’.*

The GP practice felt familiar to participants, contrasting with hospital environments that felt more threatening and brought back memories of cancer treatments and “being a patient.”

Some participants appreciated the practice nurse’s involvement for being already known, trusted, and easy to talk to; familiar with their medical background and personal situation; and able to offer longer consultations than GPs.

#### Theme 2: Content of sessions and execution of PA (program-specific domain)

Almost all participants preferred face-to-face sessions over digital or telephone sessions because it felt easier to open up and ask questions. Some participants also felt committed to do their best because of the sessions. Others wanted more frequency sessions (e.g., every 3 weeks).

Patient 1, Male, 58Y: *It functions as a big stick. Because she* [the practice nurse] *said that I had to come to her several times* […] *then I can’t just sit around and do nothing. I can’t come to her and say ‘well, I’m sorry, but I haven’t done anything all week’.*

Many appreciated the practice nurse’s role as a sounding board for sharing their story and well-being, although one participant wanted the practice nurse to talk about their disease history. Some appreciated that the practice nurse also discussed other lifestyle factors, but others did not have the same experience and suggested the need to incorporate coaching for other lifestyle domains, such as diet. The value of the practice nurse for signaling problems and referring patients to other health care professionals was also valued. One participant did not receive knowledge from the practice nurse about the specific benefits of PA for cancer. In addition, some participants experienced little guidance or coaching from the practice nurse about the options for PA.

Patient 3, Female, 45Y: *What I still miss is coaching to get more physically active. That is left entirely to your own initiative […] what you do in daily life, but also to see how that can be improved […] what kind of activities could we do, or where you could sign up to do something.*

Several participants valued that they could perform the PA alone, but some others wanted a group approach because they had positive experiences of group meetings before.

Patient 14, Female, 79Y:* I can walk, but I walk very slowly. At the neighborhood association they will start this* [group walks] *too, but I won’t be able to join because I have a very short stride. And then everybody has to wait for me to arrive. So, if I go walking, I would rather do it alone.*

Some participants had expected the program to be more intensive, with one experiencing the program and related goal setting as too focused on walking and citing that as a reason to quit. One suggestion was to use a diary to register PA instead of the number of steps from the activity tracker. Other barriers to PA were mentioned, such as pain, edema, fatigue, or a loss of confidence in one’s own body or ability.

Patient 12, Female, 67Y: *I had surgery on my kidney […] I’ve always held pain in that side, so when I’m leaning forward, I feel it* […] *Well, then you have to go up again and then, yes, then you do less, I think. You are more active when you are not bothered.*

A physically active job that involved a lot of walking facilitated PA in one participant. In another, exercising with their partner stimulated them to walk further. Several also mentioned the benefit of good outdoor conditions, including good weather and a natural environment, as a stimulus for going for a walk or a bike ride instead of taking the bus. Cold weather, rainy days, or being dark outside were also seen as hindrances to walking. Finally, a busy life with little free time limited one participant from being more active, while another was limited because her husband got sick, which was a reason for her to quit the program.

#### Theme 3: Activity tracker and goal setting (program-specific domain)

The activity tracker provided insight into daily PA levels and motivated many participants to reach their PA goals.

Patient 9, Male, 59Y: *After dinner, I looked at how many steps I had done during the day* […] *I then had an indication of ‘I have to be at this number of steps, and it’s too low’* […] *I would do a bigger walk in the evening to reach my target.*

Some mentioned that the activity tracker was not always representative of the actual PA performed, or that it was inconvenient or not allowed at work. One reported that the constant data tracking sometimes made her feel constricted.

Patient 8, Female, 55Y: *And, yes, I think constricted is a good word* […] *that all is registered* […] *as if you have to do something with it, or something like that* […] *Because I do not sleep very well* [and] *you are confronted with that or something.*

One participant mentioned he did not feel comfortable wearing the activity tracker because he was not used to wearing accessories on his hands or fingers. This led to him quitting the program.

Many participants regarded setting their own goals as a motivator for increasing PA levels. Goal setting was considered an essential element in the PA program for some.

Patient 14, Female, 79Y: *I think this was a big stick. Those goals. Then you have to* [stand up and do something]*. Because otherwise, what would you do it for?*

For some, goal setting had little effect and did not compel adherence. Another participant mentioned that wearing the activity tracker and not reaching their goal demotivated her and made her nervous or feel like a failure, which led to her quitting the program.

Patient 15, Female, 61Y: In the beginning it [activity tracker] did motivate me, but when I didn’t reach my goals, I wasn’t motivated anymore. That was a bit counterproductive for me*.* […] *The longer I was busy with that* [goal setting]*, the more nervous I became.* […] *It felt like a failure.*

Some participants experienced the frequency of goal setting and recorded PA levels as too limited. Therefore, these recommended more frequent check-ups of recorded goals and PA levels (e.g., once every week).

#### Theme 4: Experienced benefits (individual domain)

Several participants pointed out that they had gained more knowledge of the advantages of PA or had become more aware of its importance for health.

Altered PA behaviors had become a routine part of many participants’ daily lives. For example, they gave more priority to PA, were more physically active, maintained goal setting in daily life, or looked at alternatives to being physically active.

Patient 9, Male, 59Y: *What did it* [the PA program] *bring me? More discipline in being physically active* […] *I have been doing that* [walking] *more structurally because of this program. You expand the walk circuit every time … Push yourself to do more … that is what I have experienced.*

Behavioral changes were also mentioned in other lifestyle domains, such as making healthier choices regarding diet. Some also kept a closer eye on their weight, and one mentioned reducing his alcohol intake following advice from the practice nurse. However, several commented that the PA program had not added value because they were happy with their lives before participation and did not make or notice any changes in behavior.

Favorable physical and mental health outcomes were noted. For example, participants reported that they not only had more energy, better endurance, and greater flexibility but that they could also set their negative emotions aside more easily during their walks. Some also regained their trust and confidence in their own bodies during participation.

Patient 6, Female, 65Y: *I have become more fit because of it* [the PA program]*. More energy, more fitness* […] *Now, I will go out to visit someone in the evenings, because I didn’t do that in that period* [immediately after treatment]*. I just couldn’t do it.*

Participation had a positive influence of social environments. One participant mentioned that his partner had become more physically active, while another mentioned that a friend had also bought an activity tracker. One participant also indicated that the PA program had supported his return to work by helping him regain physical fitness sooner.

#### Theme 5: Personalized care needs (individual domain)

In some instances, the PA program was offered at the right time (i.e., during early recovery). These participants experienced the PA program as the closing chapter of their cancer trajectory or as a new beginning. For others, the PA program was offered too late (i.e., they wished they could have participated earlier). Almost all participants mentioned that the best timing would differ with the individual and their own treatment and recovery. Although most recommended offering the program immediately after cancer treatment, some thought that it could be offered during cancer treatment as well.

Several participants elaborated on their care needs following cancer treatment, expressing a wish to receive psychological support. They mentioned they would like to talk about their feelings, the impact of disease and its treatment, and how to get their lives back on track.

Patient 16, Male, 69Y: *Physical activity is good, but the emotional side is also very important* […] *My wife has her emotions, the kids have their emotions, and they have gone through a horrible time* […] *that upset me.*

## Discussion

### Summary

Most of the included cancer survivors valued the PA program being offered at their GP practice because it was nearby, did not involve additional costs, and felt familiar. Practice nurse consultations were valued because they acted as a sounding board for patients and allowed more time than a GP consultation. The nurse was also valued because she was familiar with the medical background and context of each patient. Use of the activity tracker and goal setting motivated most survivors to increase their PA levels, but for some, it led to demotivation and feelings of failure. Reported benefits included behavioral change and positive health outcomes. Participants wanted care to be personalized for both the program’s timing and the provision of psychological support.

### Strengths and limitations

To our knowledge, this is the first study to have evaluated the experiences of cancer survivors taking part in a PA program at a primary care setting of a GP practice. Strengths of this study are that we used sound qualitative methods and evaluated the program from patients’ perspectives, which can help to improve future PA programs for cancer survivors. A possible limitation may be that the program was implemented as part of a study. Findings are therefore colored by some who only participated to help with the research and who had no personal goals or intention to increase PA. Therefore, our results may not reflect implementation in real-world settings. Additionally, although this study took place within Dutch primary care, we believe that its application could be beneficial to health care systems that use some form of primary care.

### Institutional domain

Program accessibility was considered important in terms of location and cost, as stressed in earlier research [19, 21, 22, 40]. Patient preference to consult a primary health care professional for lifestyle advice or psychosocial support, rather than a hospital-based medical specialist, has also been addressed before [24, 40]. A PA program from a primary care setting, such as the GP practice, could serve as a suitable follow-up from rehabilitation programs offered from secondary care, allowing patients to have continued and accessible contact with medical professionals throughout their rehabilitation journey. Involvement of primary health care professionals can offer familiarity that helps reassure patients during the recovery process, allowing room for psychological support, sharing the recovery story, and regaining trust in their own body [41]. Given patients’ preference in this study to have their medical history known and the option for referral to other medical specialists, it is crucial that the professional assisting the patient operates within a health care setting rather than in community health care or through lifestyle coaches. Patients are able to turn to primary health care professionals for other health issues too, which may prevent health-related issues in the long term. However, although patients seem to value this type of care being offered at in primary care, we should acknowledge the time constraints placed on health care professionals in the current overloaded primary health care system. This would make it challenging to offer this type of care for every cancer survivor, and moreover, not every patient may need this care. Future research on the primary health care professionals’ perspectives and experiences, including GPs and practice nurses, is needed to inform us on this matter.

### Program-specific domain

Experiences with the activity tracker and goal setting varied, and could reflect individual differences in self-efficacy and goal orientation [42]. People who are performance-oriented generally have low self-efficacy for PA and are more vulnerable to feelings of failure, shame, and self-doubt if they do not achieve a goal compared to people who are task-oriented [42]. High self-efficacy is important because it can contribute to positive experiences and increase PA levels [20, 43]. Being aware of these individual differences in goal orientation and self-efficacy can allow primary care professionals to tailor their motivational techniques for behavioral change to each patient. Health care professionals should have sufficient experience in lifestyle consultation and knowledge of cancer-related disabilities, where a lack of may limit implementation [44]. Additional training, as in this study, can be offered to increase their knowledge and capabilities in coaching patients. Also, as indicated by patients in our study, a tailored approach is important when considering alternative PAs, tailoring the PA goals to the preferred activities and providing regular feedback, knowing when to refer to other health care professionals, and considering patients who might benefit from group exercise [19, 22, 28]. Coaching sessions, upon the use of a digital application or activity tracker, are important for increasing PA adherence [20] and stimulating favorable health outcomes [4, 45] and PA behavior by behavioral change techniques of self-monitoring and planning [46, 47]. Because this PA program entails general elements of behavioral change techniques and has shown efficacy in increasing PA behavior in other patient groups as well, we believe it may also benefit other patient groups in primary care settings.

### Individual domain

Participants reported favorable changes in both physical and mental health, consistent with previous literature [48, 49]. For some, the PA program served as a way to end an intense period of illness and return to relative normality by becoming occupied with something different and feeling both heard and seen. These patients may experience their period of illness as “teachable moment”, reflecting on their lives and realizing what is important for them. This moment is fruitful for inducing behavioral change, and is worthwhile to act upon in each individual patient [50, 51] Previous literature also shows that the correct timing of the program is crucial for optimal support and can differ per individual, with some wanting it during treatment but many wanting it after treatment [19, 22].

## Conclusion

Primary care is important for continued care after cancer treatment. According to patient experiences, access to a PA program in this setting is not only valued for providing health benefits but also seems to meet an unmet need for support in picking up life during cancer recovery. This study shows that these needs can be met, provided the PA program is offered nearby, without additional costs, and with the involvement of a trusted and well-trained medical professional. Personalized care in terms of coaching, support, and timing is important.

## Data Availability

The datasets used and/or analyzed during the current study are not publicly available or available from the corresponding author on reasonable request because of the qualitative nature of the interview data and the impossibility to de-identify the data.
